# 

**Published:** 1886-12-18

**Authors:** 


### THE HOSPITAL

An Institution, Family, and Parochial Journal of Hospitals, Asylums, & all Agencies for the Care of the Sick, Criticism & News.

### CONTENTS.

**Doctors' Workshops: Poor-law Infirmaries** 191

**Words of Consolation.**—XII. Contentment - - 192

**The Classes and the Masses: A Word to the Wise.**—I. The Lessons of the Times. By the Ven. Archdeacon Farrar, D.D. - - - - 193

**Hospital Administration.**—The Financial Depression at the Metropolitan Hospitals To-day and Ten Years Ago - - - - - - 195

**A District Nurse and her Patients.** By the Matron of an East London District Nursing Society - 197

**"The Hospital" and "The Globe."** Lord Salisbury's Article - - - - - - - 198

**Notes and News.**—Miscellaneous—Legacies to the Manchester Hospitals.—Bazaars.—Vacancies.—The West London Hospital.—The *Truth* Toy Fund.—Hospital Students and their Patients.—Miss Wood on the Management of Infants and Children.—Nairn Hospital and the Sanitary Committee.—Wellington Dispensary and the Resignation of Dr. Rider.—New Cottage Hospital for Abingdon, etc. - - - - - 198

**Annotations.**—The Hospitals Week Movement—Temperance and Total Abstinence.—A Hospital for Oban - 201

**Sister Olive:** A Hospital Romance.—By W. J. Eccott 202

**Reminiscences of the Insane.** By a Mental Physician - - - - - - - - 204

**For the Children's Ward.** Seasonable Decorations 204

**Till the Doctor Comes.** VI.—Bathing - - 205

**Metropolitan Hospital Sunday Fund, 1886** - 206

**Hospitals for Lady Probationers** - - - 206

**The Children's Corner.**—Puzzles, Answers, etc. - 207

**The Hospital Charity Box.**—Prizes and Results - 207

**In- and Out-Patients' Hospital Diary** - - 208

